# Histone deacetylase inhibitors protect against cisplatin-induced acute kidney injury by activating autophagy in proximal tubular cells

**DOI:** 10.1038/s41419-018-0374-7

**Published:** 2018-02-23

**Authors:** Jing Liu, Man J. Livingston, Guie Dong, Chengyuan Tang, Yunchao Su, Guangyu Wu, Xiao-Ming Yin, Zheng Dong

**Affiliations:** 1 Department of Nephrology, The Second Xiangya Hospital, Central South University, Changsha, Hunan 410011 China; 2 Department of Cellular Biology & Anatomy, Medical College of Georgia at Augusta University and Charlie Norwood VA Medical Center, Augusta, GA 30912 USA; 3 Department of Pharmacology & Toxicology, Medical College of Georgia at Augusta University and Charlie Norwood VA Medical Center, Augusta, GA 30912 USA; 4 Department of Pathology and Laboratory Medicine, Indiana University School of Medicine, Indianapolis, IN USA

## Abstract

Histone deacetylase inhibitors (HDACi) have therapeutic effects in models of various renal diseases including acute kidney injury (AKI); however, the underlying mechanism remains unclear. Here we demonstrate that two widely tested HDACi (suberoylanilide hydroxamic acid (SAHA) and trichostatin A (TSA)) protect the kidneys in cisplatin-induced AKI by enhancing autophagy. In cultured renal proximal tubular cells, SAHA and TSA enhanced autophagy during cisplatin treatment. We further verified the protective effect of TSA against cisplatin-induced apoptosis in these cells. Notably, inhibition of autophagy by chloroquine or by autophagy gene 7 (Atg7) ablation diminished the protective effect of TSA. In mice, TSA increased autophagy in renal proximal tubules and protected against cisplatin-induced AKI. The in vivo effect of TSA was also abolished by chloroquine and by Atg7 knockout specifically from renal proximal tubules. Mechanistically, TSA stimulated AMPK and inactivated mTOR during cisplatin treatment of proximal tubule cells and kidneys in mice. Together, these results suggest that HDACi may protect kidneys by activating autophagy in proximal tubular cells.

## Introduction

Histone deacetylases (HDACs) are important players in epigenetic regulation that catalyze the removal of acetyl groups from ε-N-acetyl lysine residues on histones, resulting in an increase of positive charges in histones, tight DNA binding, and compact chromatin to repress gene transcription^1^. HDACs may also deacetylate non-histone proteins. As such, HDACs play crucial roles in diverse biological processes and deregulation of HDACs contributes to the pathogenesis of major diseases^2^. The research of HDACs has also led to the development of HDAC inhibitors (HDACis) therapeutic effects^3^. In kidneys, HDACis have therapeutic potentials in experimental models of renal fibrosis^4–6^, acute kidney injury (AKI)^7–9^, polycystic kidney disease^10,11^, diabetic nephropathy^12,13^, and HIV-associated nephropathy^14^. The mechanisms underlying the beneficial effect of HDACis in kidneys remain poorly understood^15,16^.

We demonstrated dose-dependent effects of HDACi in renal tubular cells^8,17^. At micromolar concentrations, suberoylanilide hydroxamic acid (SAHA) induced cell death in these cells, whereas at lower doses SAHA protected the cells from cisplatin-induced apoptosis. Cisplatin, a potent cancer therapy drug, has notorious side effects in normal tissues, especially in kidneys^18–22^. A pathological hallmark of cisplatin nephrotoxicity is cell injury and death in renal tubules^23^. Interestingly, HDACis, including both SAHA and trichostatin A (TSA), may suppress cisplatin-induced tubular cell apoptosis and AKI in mice^7–9^, but the underlying mechanism is largely unclear.

Autophagy is a highly conserved lysosomal degradation pathway that eliminates protein aggregates and dysfunctional organelles^24,25^. In cellular stress, autophagy is activated and serves primarily as an adaptive mechanism for cell survival, whereas deregulated autophagy plays important roles in the pathogenesis of various diseases^26,27^. In ischemic and nephrotoxic AKI, autophagy is induced to protect against tubular cell injury and death^28–31^. The current study tested the hypothesis that HDACis may protect kidney cells and tissues by upregulating autophagy. We demonstrated that both SAHA and TSA enhanced autophagy in renal tubular cells. TSA further protects against cisplatin-induced injury in renal tubular cells and AKI in mice, and notably, the protective effects of TSA were diminished by pharmacological and genetic inhibition of autophagy.

## Results

### Autophagy is induced by TSA and SAHA in renal proximal tubular cells (RPTCs)

**Fig. 1:**
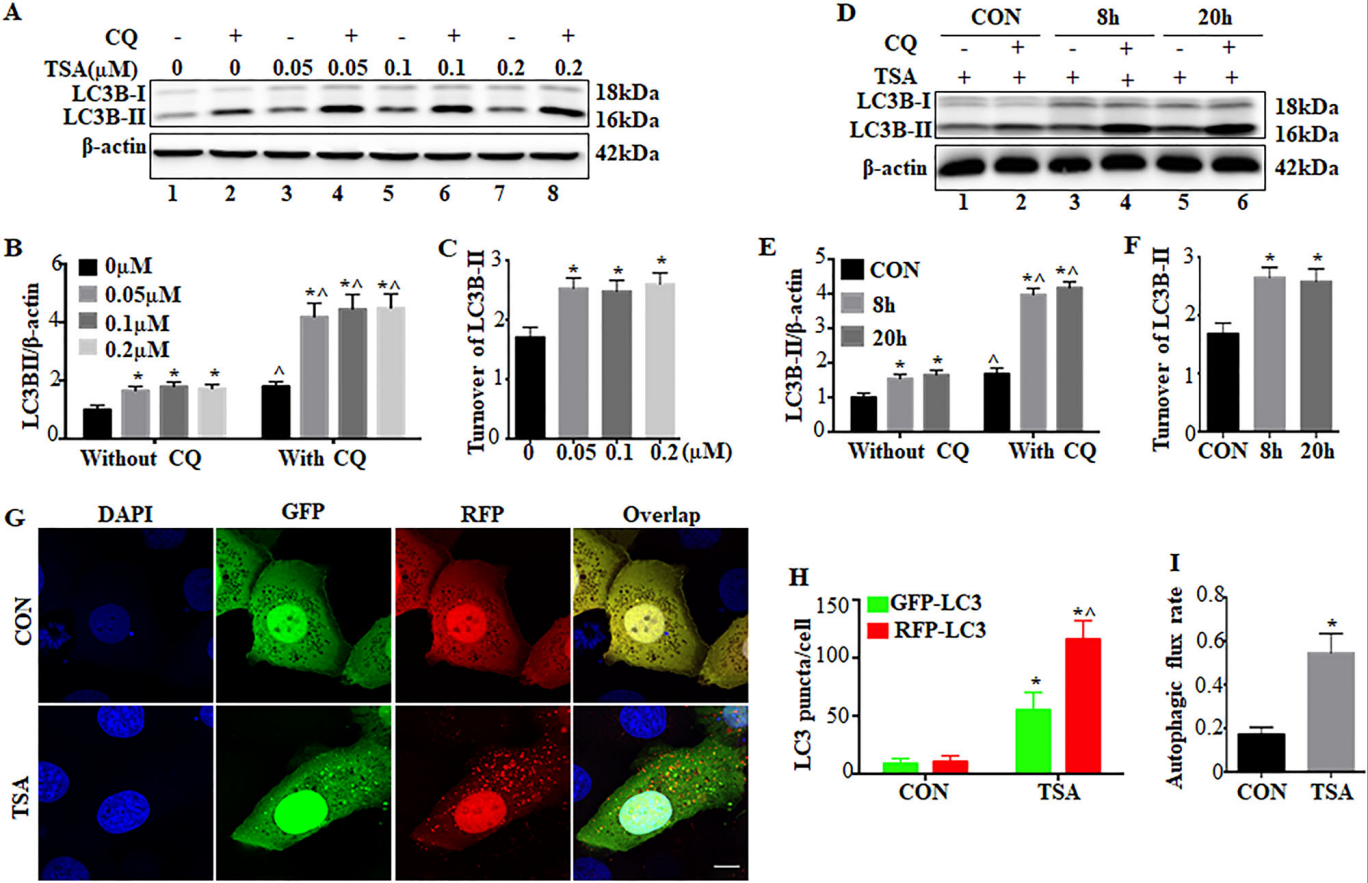
Autophagy is induced by TSA and SAHA in renal proximal tubular cells. **a**–**c** RPTC cells were incubated with at the indicated concentrations for 20 h in the absence or presence of 20 μM chloroquine. **d**–**f** RPTC cells were treated with 0.1 μM TSA for 8 h or 20 h in the absence or presence of 20 μM chloroquine. After treatment, whole-cell lysates were collected for immunoblot analysis of LC3B. β-Actin was probed as a loading control. **a**, **d** Representative immunoblot. **b**, **c**, **e**, **f** Densitometric analysis of LC3B signals. After normalization with β-actin, the protein signal of control was arbitrarily set as 1, and the signals of other conditions were normalized with control to calculate fold changes. Data are expressed as mean ± SD. *P < 0.05, significantly different from the control group. ^*P* < 0.05, significantly different from without chloroquine **g**–**i** RPTC cells were transiently transfected with mRFP-GFP-LC3 and then treated with 0.1 μM TSA for 20 h. After treatment, cells were fixed with 4% paraformaldehyde for fluorescence microscopy. **g** Representative images showing GFP-LC3 and mRFP-LC3 puncta in transfected cells (×630). Scale bar: 15 μm. **h** Quantitative analysis of GFP-LC3 and RFP-LC3 puncta. Data are expressed as mean ± SD. *P < 0.05, significantly different from the control group. ^*P* < 0.05, significantly different from GFP-LC3 puncta counting. **i** Analysis of autophagic flux rate. Data are expressed as mean ± SD. *P < 0.05, significantly different from the control group

Treatment of RPTCs with TSA led to LC3B-II accumulation, a biochemical hallmark of autophagy^32^ (Fig. 1a: lanes 3, 5, 7 vs lane 1; lanes 4, 6, 8 vs lane 2). LC3B-II accumulation was markedly increased by chloroquine, an auto-lysosomal inhibitor^32^ (Fig. 1a: lanes 2, 4, 6, 8 vs lanes 1, 3, 5, 7). The effect of chloroquine appeared more in TSA-treated cells (lane 2 vs lanes 4, 6, 8), indicating an increased LC3B-II turnover and induction of autophagic flux by TSA. We then examined the expression of LC3B-II at two different time points of 8 and 20 h. There was an early LC3B-II accumulation following 8 h of TSA treatment (Fig. 1d, lane 3 vs lane 1; lane 4 vs lane 2), accompanied with an increased LC3B-II turnover (lanes 2, 4 vs lanes 1, 3). Consistently, these changes were maintained at 20 h of TSA treatment (lanes 5, 6 vs lanes 1, 2). The immunoblotting results were confirmed by densitometry of LC3B-II signals (Fig. 1b, c, e, f). Similarly, SAHA, another pan-HDACi, also induced autophagy in RPTC cells as indicated by LC3B-II (Supplementary Figure 1). We further monitored autophagic dynamics by transfecting monomeric red fluorescent protein (mRFP)-green fluorescent protein (GFP)-LC3 plasmids. In control cells, both green GFP-LC3 and red RFP-LC3 signals were mostly diffuse in cells (Fig. 1g, CON). Following TSA treatment, numerous GFP-LC3 and RFP-LC3 puncta appeared (Fig. 1g, TSA). Almost all GFP signals co-localized with RFP, exhibiting a yellow color indicative of autophagosomes. Notably, the acid-sensitive GFP fluorescence faded or disappeared upon fusion with lysosomes, whereas the acid-insensitive RFP signals were maintained. Therefore, a portion of RFP-LC3 puncta with no or very weak GFP signals showed orange-red staining in overlapping images, which represented autolysosomes (Fig. 1g, TSA). Quantitatively, the number of autophagosomes per cell was increased from ~9 in control cells to ~55 in TSA-treated cells. The number of autolysosomes per cell was also remarkably elevated from ~2 in control to ~61 in TSA-treated cells (Fig. 1h). We further calculated the proportion of autolysosomes in the total number of RFP-LC3 puncta to indicate autophagic flux^32^. Control cells had a basal rate of ~17% autophagic flux, which was increased to ~54% by TSA (Fig. 1i).

### TSA enhances autophagy during cisplatin treatment of proximal tubular cells

**Fig. 2:**
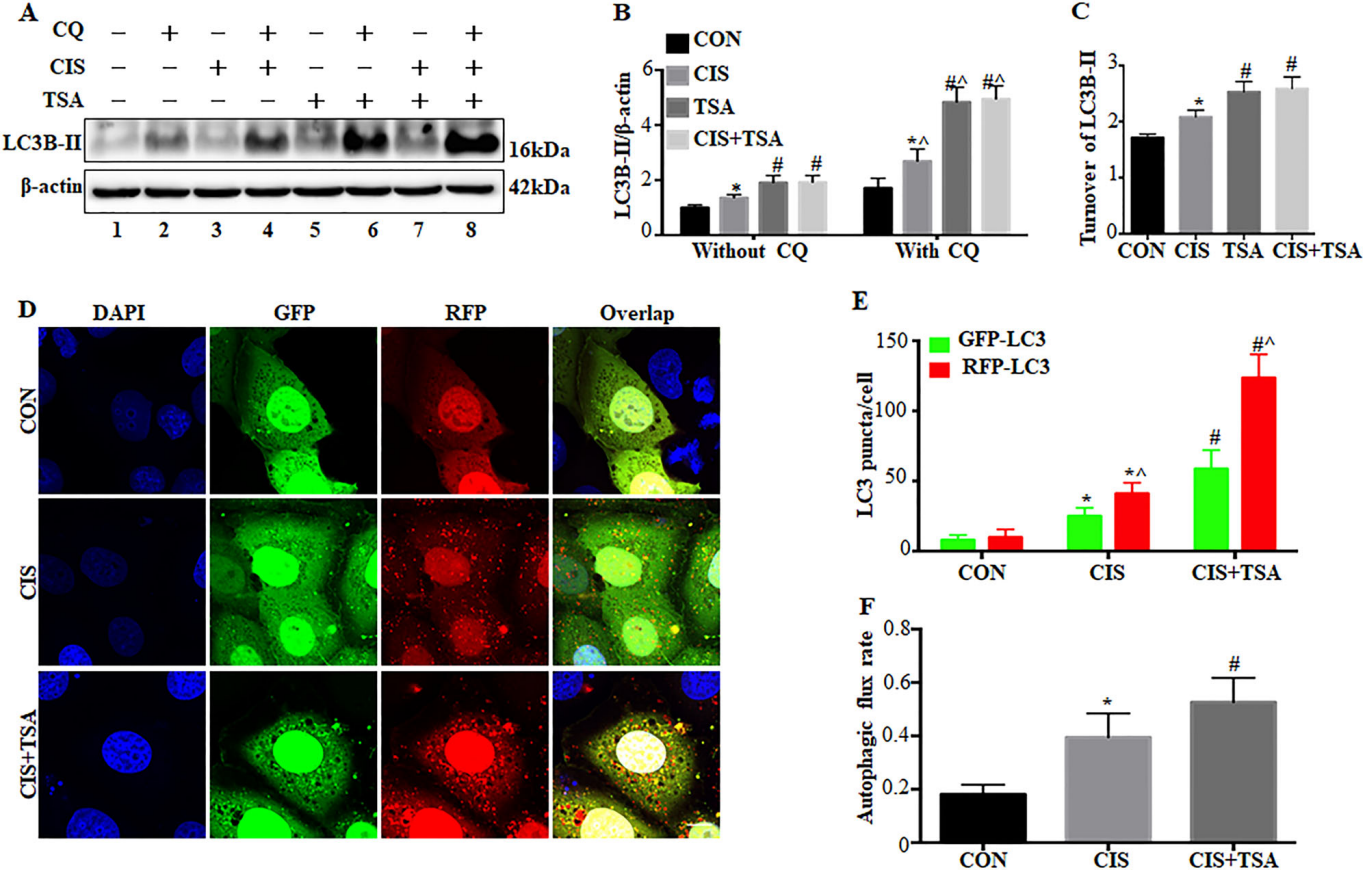
TSA enhances autophagy during cisplatin treatment of renal proximal tubular cells. **a**–**c** RPTC cells were untreated or treated for 20 h with cisplatin (20 μM), TSA (0.1 μM), or cisplatin+TSA in the absence or presence of 20 μM chloroquine. The cells were then collected for immunoblot analysis of LC3B. β-Actin was used as a loading control. **a** Representative immunoblots. **b**, **c** Densitometric analysis of LC3B signals. Data are expressed as mean ± SD. *P < 0.05, significantly different from the control group. ^#^*P* < 0.05, significantly different from the cisplatin-only group. ^*P* < 0.05, significantly different from the corresponding group without chloroquine. **d**–**f** RPTC cells were transiently transfected with mRFP-GFP-LC3 and then treated as indicated. After treatment, cells were fixed with 4% paraformaldehyde for fluorescence microscopy. **d** Representative images showing GFP-LC3 and mRFP-LC3 puncta in transfected cells (×630). Scale bar: 15 μm. **e** Quantitative analysis of GFP-LC3 and RFP-LC3 puncta. Data are expressed as mean ± SD. *P < 0.05, significantly different from the control group. ^#^*P* < 0.05, significantly different from the cisplatin-only group. ^*P* < 0.05, significantly different from GFP-LC3 puncta counting. **f** Analysis of autophagic flux rate. Data are expressed as mean ± SD. *P < 0.05, significantly different from control group. ^#^*P* < 0.05, significantly different from the cisplatin-only group

We further examined the effects of TSA on cisplatin-induced autophagy in RPTC cells. Cisplatin treatment for 20 h led to a moderate yet significant accumulation of LC3B-II (Fig. 2a, b: lanes 3, 4 vs lanes 1, 2), which was further enhanced by TSA (lanes 7, 8 vs lanes 3, 4). LC3B-II turnover assay in Fig. 2c also showed that control cells had a 1.68-fold basal level of turnover, which was increased in cisplatin-treated cells (2.08-fold) but significantly promoted by cisplatin+TSA (2.6-fold). In the cells expressing mRFP-GFP-LC3 (Fig. 2d–f), cisplatin treatment increased the numbers of both GFP-LC3 and RFP-LC3 puncta. Notably, the average numbers of autophagosomes and autolysosomes per cell in cisplatin-only group were ~25 and ~16, which were increased to ~58 and ~65, respectively, in the cisplatin+TSA group (Fig. 2e). Consistently, cisplatin-induced autophagic flux was also elevated from ~39% to ~53% by TSA (Fig. 2f), indicating that TSA further enhances cisplatin-induced autophagy in RPTC cells.

### Inhibition of autophagy by chloroquine eliminates the protective effects of TSA in cisplatin-treated RPTC cells

**Fig. 3:**
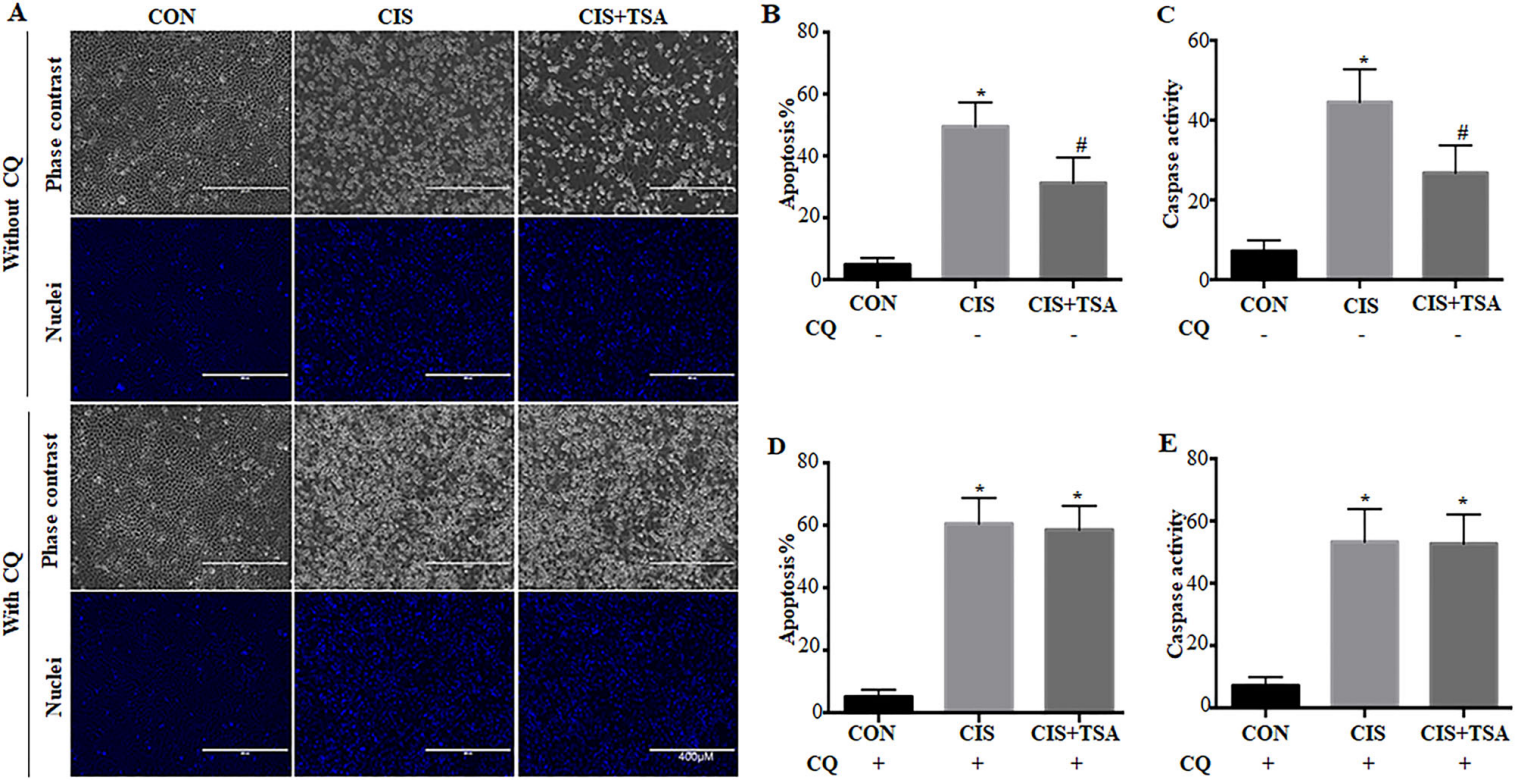
Inhibition of autophagy by chloroquine eliminates the protective effects of TSA in cisplatin-treated RPTC cells. RPTC cells were untreated or treated with cisplatin (20 μM) and cisplatin+TSA (0.1 μM) in the absence or presence of 20 μM chloroquine for 20 h. **a** Representative images of cellular and nuclear morphology (×100). Scale bar: 400 μm. **b**, **d** Quantification of apoptosis percentage. **c**, **e** Caspase activity measured by enzymatic assays using DEVD-AFC as substrates. Data in **b**–**e** are expressed as mean ± SD. *P < 0.05, significantly different from the control group. ^#^*P* < 0.05, significantly different from the cisplatin-only group

To determine the involvement of autophagy in the protective effect of HDACi, we first compared the effects of TSA on cisplatin-induced tubular cell apoptosis in the absence vs presence of chloroquine. In the absence of chloroquine, cisplatin treatment for 20 h led to ~50% apoptosis in RPTC cells, which was partially but significantly reduced to ~30% by TSA (Fig. 3a: without CQ, Fig. 3b). Consistently, cisplatin-induced caspase activation was also attenuated by TSA under these conditions (Fig. 3c). In sharp contrast, the beneficial role of TSA disappeared when autophagy was inhibited by chloroquine. Consistent with our previous studies^33,34^, cisplatin-induced apoptosis in RPTC cells was worsened by chloroquine. Notably, in the presence of chloroquine, TSA was unable to suppress cisplatin-induced apoptosis and caspase activation (Fig. 3a: with CQ, Fig. 3d, e), suggesting a role of autophagy in the cytoprotective effects of TSA.

### The cytoprotective effect of TSA is abrogated in *Atg7*-KO mouse kidney proximal tubular cells

**Fig. 4:**
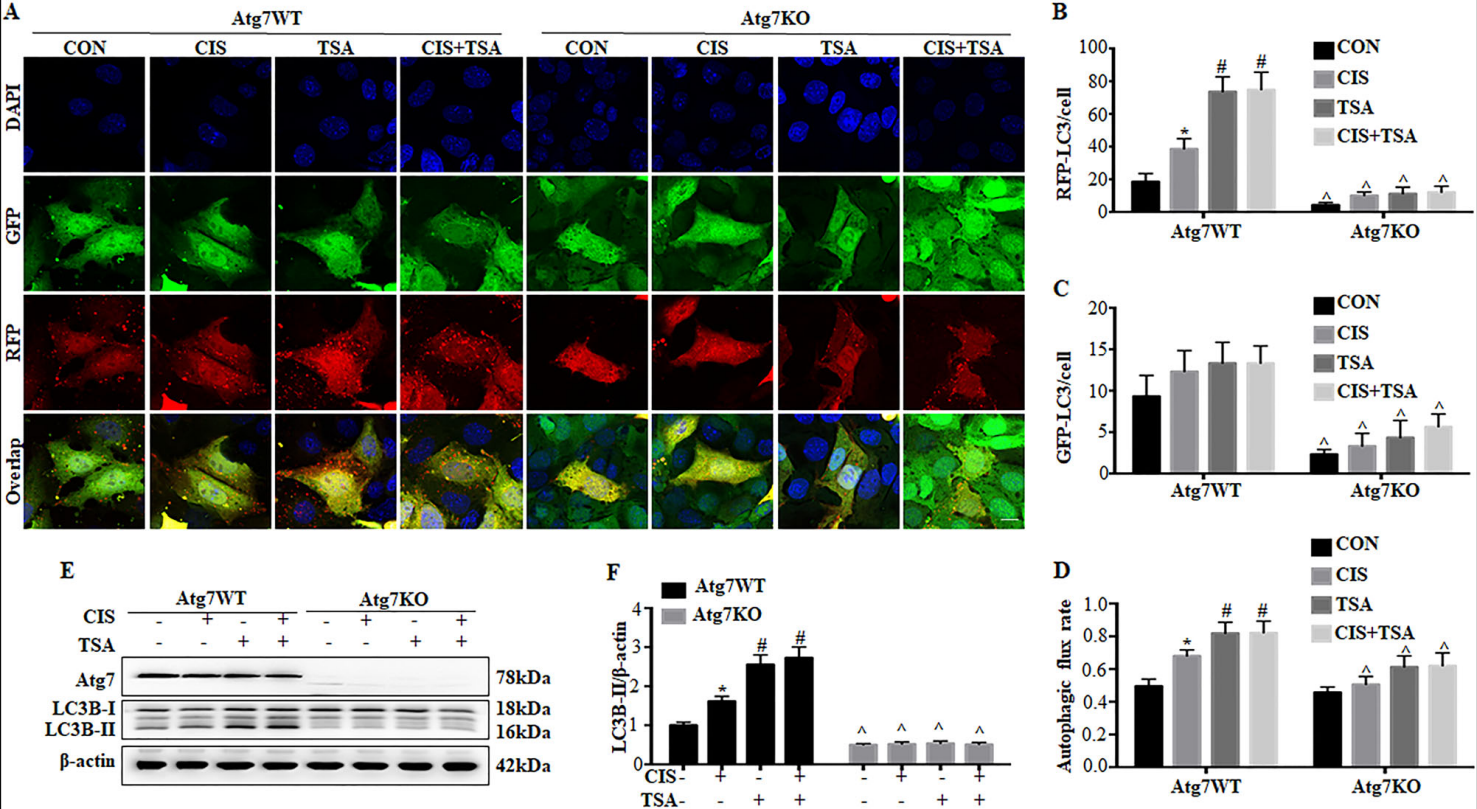
Autophagy is impaired in *Atg7*-KO mouse kidney proximal tubular cells. Wild-type (*Atg7* WT) and *Atg7*-knockout (*Atg7* KO) MPTC cells were transiently transfected with mRFP-GFP-LC3 and then untreated or treated with cisplatin (20 μM), TSA (0.1 μM), or cisplatin+TSA for 20 h. **a** Representative images showing GFP-LC3 and mRFP-LC3 puncta (×630). Scale bar: 15 μm. **b**, **c** Quantitative analysis of GFP-LC3 puncta and RFP-LC3 puncta. **d** Analysis of autophagic flux rate. Data in **b**, **c**, **d** are expressed as mean ± SD. *P < 0.05, significantly different from the control group. ^#^*P* < 0.05, significantly different from the cisplatin-only group. ^*P < *0.05, significantly different from the corresponding group in *Atg7* WT cells. **e**, **f** *Atg7* WT and *Atg7* KO MPTC cells were treated as described above without transfection. Whole-cell lysates were collected after treatment for immunoblot analysis of LC3B and ATG7. β-Actin was used as a loading control. **e** Representative immunoblots. **f** Densitometric analysis of LC3B signals. Data are expressed as mean ± SD. *P < 0.05, significantly different from the control group. ^#^*P* < 0.05, significantly different from the cisplatin-only group. ^*P < *0.05, significantly different from the corresponding group in *Atg7* WT cells

We further compared the effects of TSA in *Atg7*-KO and wild-type (WT) mouse proximal tubular cells (MPTCs) generated in our recent work^35^. We verified autophagy deficiency in *Atg7-*KO cells by transfecting mRFP-GFP-LC3 (Fig. 4a–d). In control WT cells. cisplatin induced the formation of autophagosomes and autolysosomes and an increase of autophagic flux. TSA activated autophagy on its own, and importantly, it further promoted cisplatin-induced autophagy in WT MPTC cells (*Atg7* WT, TSA, CIS+TSA). However, these autophagic responses were remarkably impaired in *Atg7*-KO MPTC cells (Fig. 4a–d, *Atg7* KO). GFP-LC3 puncta were rarely seen in these knockout (KO) cells. Under both control and cisplatin-treated conditions, the numbers of RFP-LC3 puncta and autophagic flux rate were largely reduced in *Atg7* KO cells as compared with WT cells. Furthermore, the autophagy-stimulating effects of TSA (either used alone or with cisplatin) were also abrogated in *Atg7* KO cells. These morphological observations were further confirmed by immunoblot analysis of ATG7 and LC3B (Fig. 4e, f). In WT cells, cisplatin induced a ~1.7-fold LC3B-II accumulation, which was further increased to ~2.7-fold by TSA. By contrast, there was no detectable ATG7 expression in *Atg7* KO cells, and as a result, the conversion of LC3B-I to LC3B-II was almost completely attenuated.

**Fig. 5:**
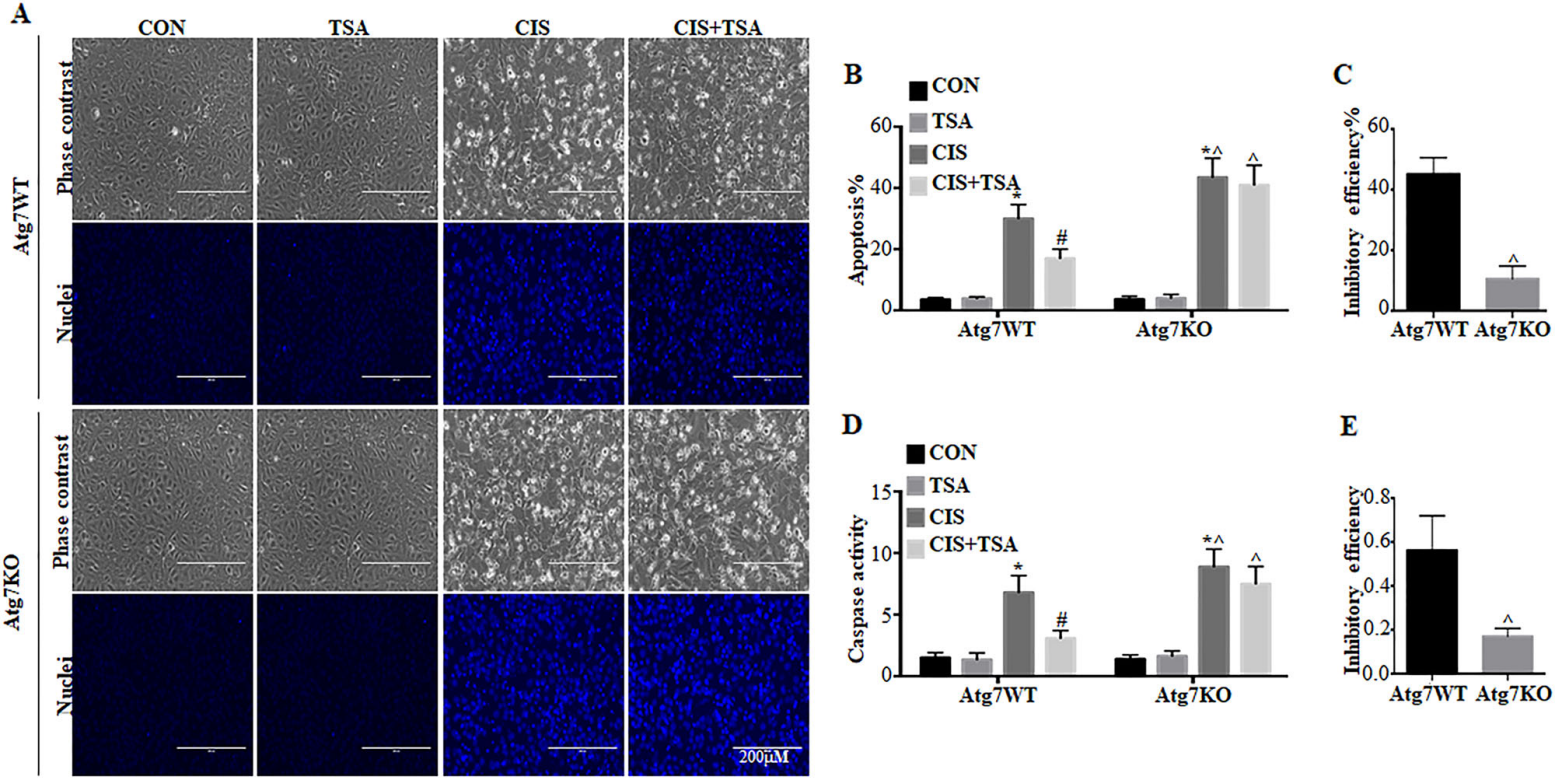
The cytoprotective effect of TSA is abrogated in *Atg7*-KO mouse kidney proximal tubular cells. *Atg7* WT and *Atg7* KO MPTC cells were untreated or treated with cisplatin (20 μM), TSA (0.1 μM), or cisplatin+TSA for 20 h. After treatment, cells were collected for morphological and biochemical analysis. **a** Representative images of cellular and nuclear morphology of apoptosis (×200). Scale bar: 200 μm. **b** Quantification of apoptosis percentage. **c** Efficiency of apoptosis inhibition by TSA (*Atg7*-WT vs *Atg7*-KO). **d** Caspase activity measured by enzymatic assays. **e** Efficiency of TSA reducing caspase activation (cisplatin only vs cisplatin+TSA). Data in **b**–**e** are expressed as mean ± SD. *P < 0.05, significantly different from the control group. ^#^*P* < 0.05, significantly different from the cisplatin-only group. ^*P < *0.05, significantly different from the corresponding group in *Atg7* WT cells

We next examined the effects of autophagy deficiency in *Atg7* KO MPTC cells on cisplatin-induced apoptosis. Both WT and Atg7-KO cells had minimal apoptosis under control conditions or TSA-only treatment (Fig. 5a, b). Cisplatin induced ~30% apoptosis in WT cells, which was reduced to ~16% by TSA. Notably, cisplatin induced ~46% apoptosis in *Atg7* KO cells, which was only slightly suppressed by TSA to 41% (Fig. 5a, b), indicating the protective effects of TSA were abolished in *Atg7* KO cells. By calculation, the inhibitory efficiency of TSA was 46% in WT cells and 11% in ATG7 KO cells (Fig. 5c). Consistently, TSA significantly suppressed caspase activation during cisplatin treatment in WT cell, but it was much less effective in *Atg7-*KO cells (Fig. 5d, e). Together with the choloquine results, these data provide convincing evidence that autophagy plays a critical role in the cytoprotective role of TSA.

### TSA enhances renal tubular autophagy during cisplatin treatment in mice

**Fig. 6:**
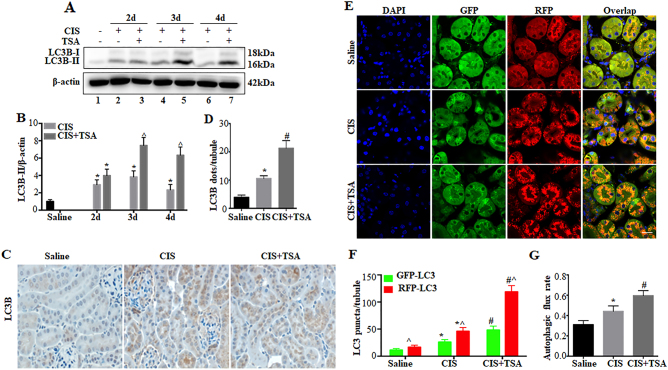
TSA enhances renal tubular autophagy during cisplatin treatment in mice. C57BL/6 mice were injected with saline or a single dose of cisplatin (30 mg/kg, i.p.) in the absence (−) or presence (+) of TSA (1 mg/kg, i.p., daily injection) (*n* = 7 for saline control, *n* = 18 for cisplatin-only, *n* = 20 for cisplatin+TSA). **a**, **b** Kidneys were collected at the indicated time points for immunoblot analysis of LC3B. β-Actin was used as a loading control. **a** Representative immunoblots. **b** Densitometric analysis of LC3B signals. Data are expressed as mean ± SD. *P < 0.05, significantly different from the saline group. ^*P < *0.05, significantly different from the cisplatin-only group. **c**, **d** Kidneys were collected 3 days after treatment for immunohistochemical staining of LC3B. **c** Representative images of LC3B staining (×400). Scale bar: 15 μm. **d** Quantitative analysis of punctate LC3B staining. Data are expressed as mean ± SD. *P < 0.05, significantly different from the saline group. ^#^*P* < 0.05, significantly different from the cisplatin-only group. **e**–**g** CAG-RFP-GFP-LC3 mice were injected with saline or a single dose of cisplatin (30 mg/kg, i.p.) only or cisplatin+TSA (1 mg/kg, i.p. daily) (*n* = 3 for saline control, *n* = 3 for cisplatin-only, *n* = 3 for cisplatin+TSA). Kidneys were collected 3 days after treatment for fluorescence microscopy. **e** Representative images showing GFP-LC3 and RFP-LC3 puncta in renal tubules of CAG-RFP-GFP-LC3 mice (×630). Scale bar: 15 μm. **f** Quantitative analysis of GFP-LC3 puncta and RFP-LC3 puncta. Data are expressed as mean ± SD. *P < 0.05, significantly different from the saline group. ^#^*P* < 0.05, significantly different from the cisplatin-only group. ^*P < *0.05, significantly different from GFP-LC3 puncta counting. **g** Analysis of autophagic flux rate. Data are expressed as mean ± SD. *P < 0.05, significantly different from the saline group. ^#^*P* < 0.05, significantly different from the cisplatin-only group

We then examined the effects of TSA on tubular autophagy in a mouse model of cisplatin-induced AKI. As previously^33,34^, a single dose of cisplatin at 30 mg/kg induced a persistent upregulation of LC3B-II in kidneys for up to 4 days (Fig. 6a: lanes 2, 4, 6 vs lane 1). TSA daily injection at 1 mg/kg further enhanced LC3B-II accumulation in cisplatin-treated C57Bl/6 mice (Fig. 6a: lanes 3, 5, 7 vs lanes 2, 4, 6). These results were substantiated by densitometric analysis of LC3B-II signals (Fig. 6b). Autophagy induction by cisplatin was further shown by immunohistochemical staining of LC3B (Fig. 6c), which showed a granular, punctate staining in proximal tubule cells during cisplatin treatment. Of note, TSA further enhanced LC3B punctate staining in both numbers and intensity. Quantitatively, the number of LC3B-positive puncta per proximal tubule was increased from ~4 in saline control kidneys to ~10 in cisplatin-treated mice and further to ~23 in the cisplatin+TSA group (Fig. 6d). To determine the dynamic changes of autophagy in kidneys, we tested the autophagy reporter mouse model that expresses the CAG-RFP-GFP-LC3 transgene^36^. As shown in Fig. 6e, the majority of renal tubules in control kidneys had few GFP-LC3 puncta, while a small percentage of tubules had RFP-LC3 puncta at relatively low intensity at the apical side toward the lumen. Cisplatin treatment led to increases in the numbers of both GFP-LC3 and RFP-LC3 puncta in proximal tubules, which were further increased by TSA. Under these conditions, the punctate LC3 staining, particularly RFP-LC3 puncta, accumulated intensively around the nuclei in proximal tubular cells (Fig. 6e). We further calculated the numbers of autophagosomes and autolysosomes as well as autophagic flux rate in vivo (Fig. 6f, g). Saline control mice had ~11 autophagosomes (GFP-LC3 and RFP-LC3 co-staining puncta) per proximal tubule, which was increased to ~26 by cisplatin and further to ~48 by cisplatin+TSA. The number of autolysosomes (RFP-LC3 puncta without GFP-LC3 signal) per proximal tubule was also increased from ~6 in saline control to ~21 in cisplatin-treated kidneys and further to ~70 in the cisplatin+TSA group (Fig. 6f). Accordingly, autophagic flux rate was increased from ~31% in saline control to ~44% by cisplatin and further to ~59% by cisplatin+TSA (Fig. 6g). Together, these results suggest that TSA promotes cisplatin-induced autophagy in proximal tubules in mice.

### Inhibition of autophagy by chloroquine abolishes the protective effects of TSA in cisplatin-induced AKI in mice

**Fig. 7:**
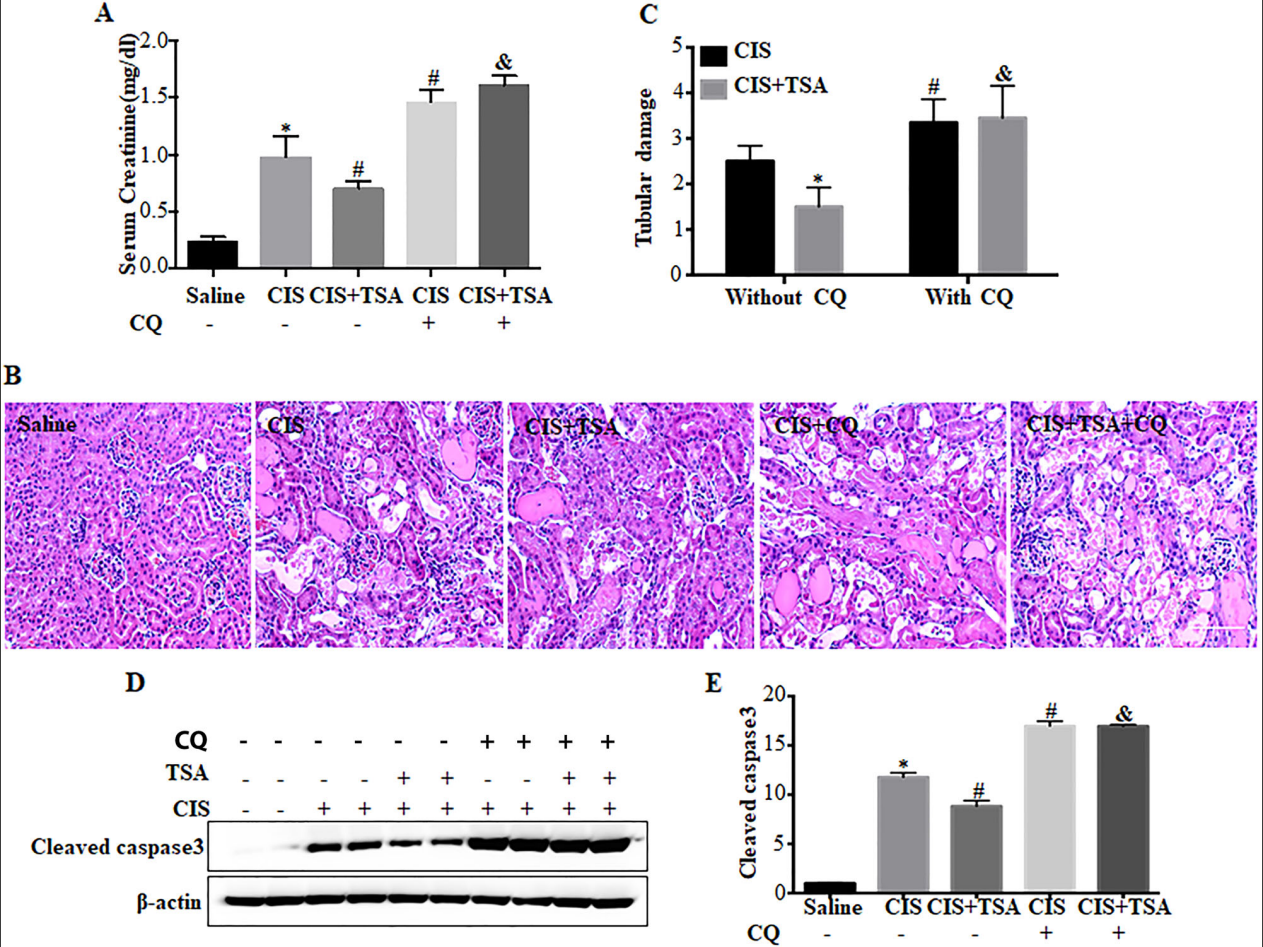
Chloroquine abolishes the protective effects of TSA in cisplatin-induced AKI in mice. C57BL/6 mice were divided into five groups for the following treatment: (1) saline (*n* = 8), (2) a single dose of cisplatin (30 mg/kg, i.p.) only (*n* = 12), (3) cisplatin+TSA (1 mg/kg, i.p., daily) (*n* = 15), (4) cisplatin+chloroquine (60 mg/kg, i.p., daily) (*n* = 6), and (5) cisplatin+TSA+chloroquine (*n* = 7). Blood samples and kidneys were collected 3 days after treatment for biochemical and histological analyses. **a** Serum creatinine. **b** Representative images of kidney H–E staining (×200). Scale bar: 100 μm. **c** Pathological score of tubular damage. Data in **a** and **c** are expressed as mean ± SD. *P < 0.05, significantly different from the saline group. ^#^*P* < 0.05, significantly different from the cisplatin-only group. ^&^*P*<   0.05, significantly different from the cisplatin+TSA group. **d** Representative immunoblots of cleaved caspase 3. **e** Densitometric analysis of cleaved caspase 3 signals. Data are expressed as mean ± SD. *P < 0.05, significantly different from the saline group. ^#^*P < *0.05, significantly different from the cisplatin-only group. ^&^*P < *0.05, significantly different from the cisplatin with the TSA group

To delineate the role of autophagy in vivo, we examined chloroquine on the renoprotective effects of TSA in cisplatin-treated C57Bl/6 mice. Cisplatin treatment led to AKI as indicated by increased serum creatinine (Fig. 7a). TSA suppressed cisplatin-induced serum creatinine from ~1.1 to ~0.7 mg/dl (CIS vs CIS+TSA). Consistenly, cisplatin-induced tubular damage in renal cortex and out medulla was also inhibited by TSA. Hematoxylin–eosin (H–E) staining revealed that cisplatin induced histopathological damages of kidney tissues, which were ameliorated by TSA (Fig. 7b: CIS vs CIS+TSA). In quantification, tubular damage score was reduced from ~3 in the cisplatin group to ~2 in the cisplatin+TSA group (Fig. 7c: CIS vs CIS+TSA). Cisplatin also induced caspase activation and tubular apoptosis as indicated by cleaved caspase 3, which was also attenuated by TSA (Fig. 7d: CIS vs CIS+TSA). However, in the presence of chloroquine, the protective effects of TSA were completely compromised (Fig. 7a–d: CIS+CQ vs CIS+TSA+CQ), indicating that autophagy induction is involved in the renoprotective effects of TSA.

### The renoprotective effect of TSA in cisplatin-induced AKI is lost in PT-*Atg7* KO mice

**Fig. 8:**
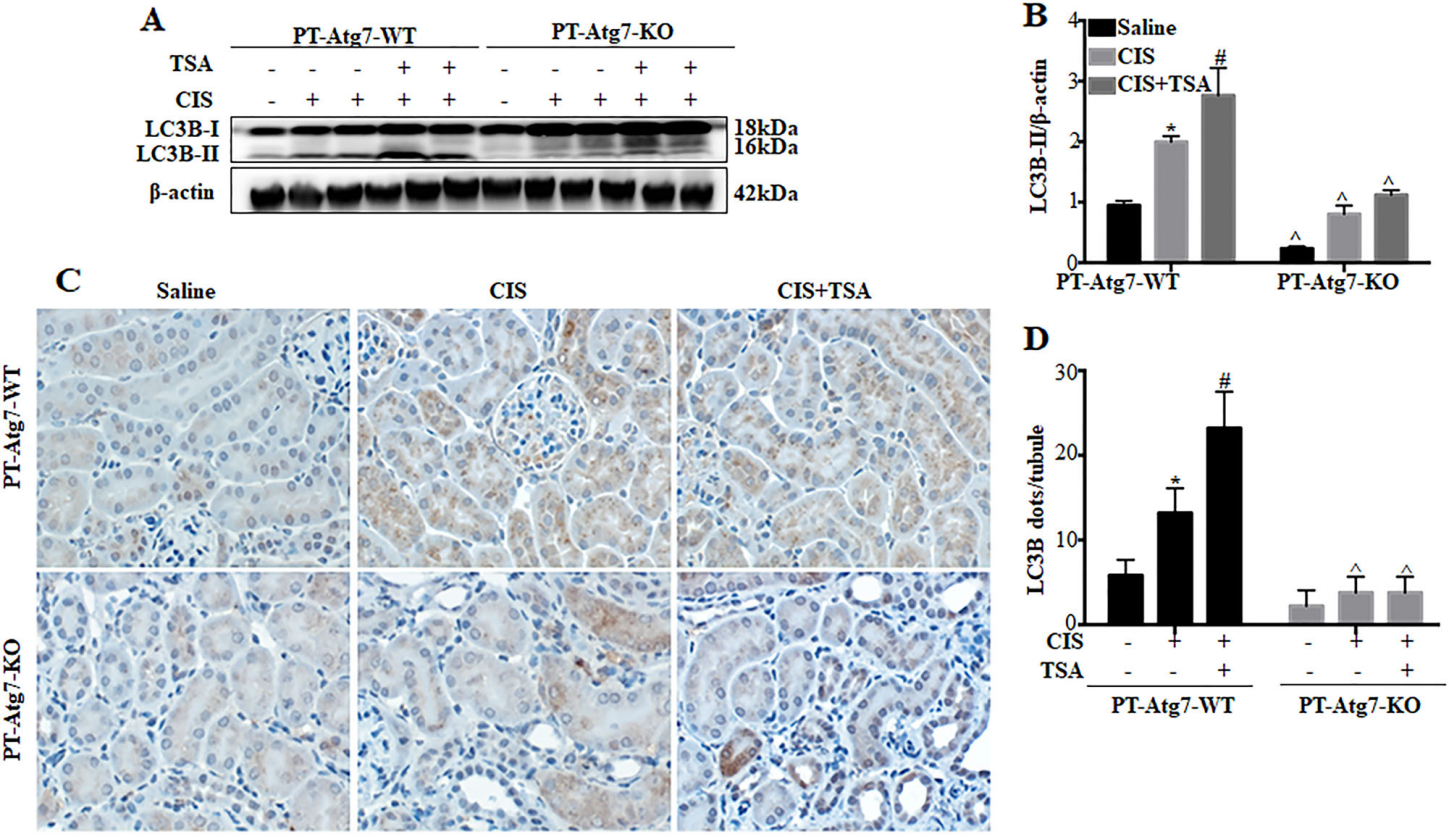
Autophagy is defective in renal proximal tubules of PT-*Atg7* KO mice. Wild-type (PT-*Atg7* WT) and PT-*Atg7* KO mice were injected with saline or a single dose of cisplatin (30 mg/kg, i.p.) in the absence (−) or presence (+) of TSA (1 mg/kg, i.p., daily injection) (*n* = 5–6 for each condition). Kidneys were collected 3 days after treatment for immunoblot analysis and immunohistochemical staining of LC3B. **a** Representative immunoblots. β-Actin was loading control. **b** Densitometric analysis of LC3B signals. **c** Representative images of LC3B immunohistochemical staining (×400). Scale bar: 15 μm. **d** Quantitative analysis of punctate LC3B staining. Data in **b** and **d** are expressed as mean ± SD. *P < 0.05, significantly different from the saline group. ^#^*P* < 0.05, significantly different from the cisplatin-only group. ^*P* < 0.05, significantly different from the corresponding groups of PT-*Atg7* WT mice

We further determined whether the renoprotective effect of TSA depends on autophagy by testing the conditional autophagy-deficient mouse model with *Atg7* specifically deleted from kidney proximal tubules (PT-*Atg7* KO)^34,35^. In WT control (PT-*Atg7* WT) mice, cisplatin led to LC3B-II accumulation in kidney tissues on day 3. This upregulation of LC3B-II was further enhanced by TSA (Fig. 8a, b: PT-*Atg7* WT). In contrast, the accumulation of LC3B-II were largely inhibited in PT-*Atg7* KO kidneys, indicating autophagy deficiency in these mice (Fig. 8a, b: PT-*Atg7* KO). In immunohistochemical analysis, cisplatin induced a punctate LC3B staining in proximal tubules and the intensity of LC3B-positive puncta was further elevated by TSA in PT-*Atg7* WT mice (Fig. 8c: PT-*Atg7* WT). Quantitatively, in PT-*Atg7* WT mice there were ~6 LC3B-positive puncta per tubule in the saline control group, which was increased to ~13 by cisplatin and further to ~23 in the cisplatin+TSA group (Fig. 8d: PT-*Atg7* WT). In contrast, PT-*Atg7* KO mice mainly showed diffuse LC3B staining in proximal tubules (Fig. 8c, d: PT-*Atg7* KO).

**Fig. 9:**
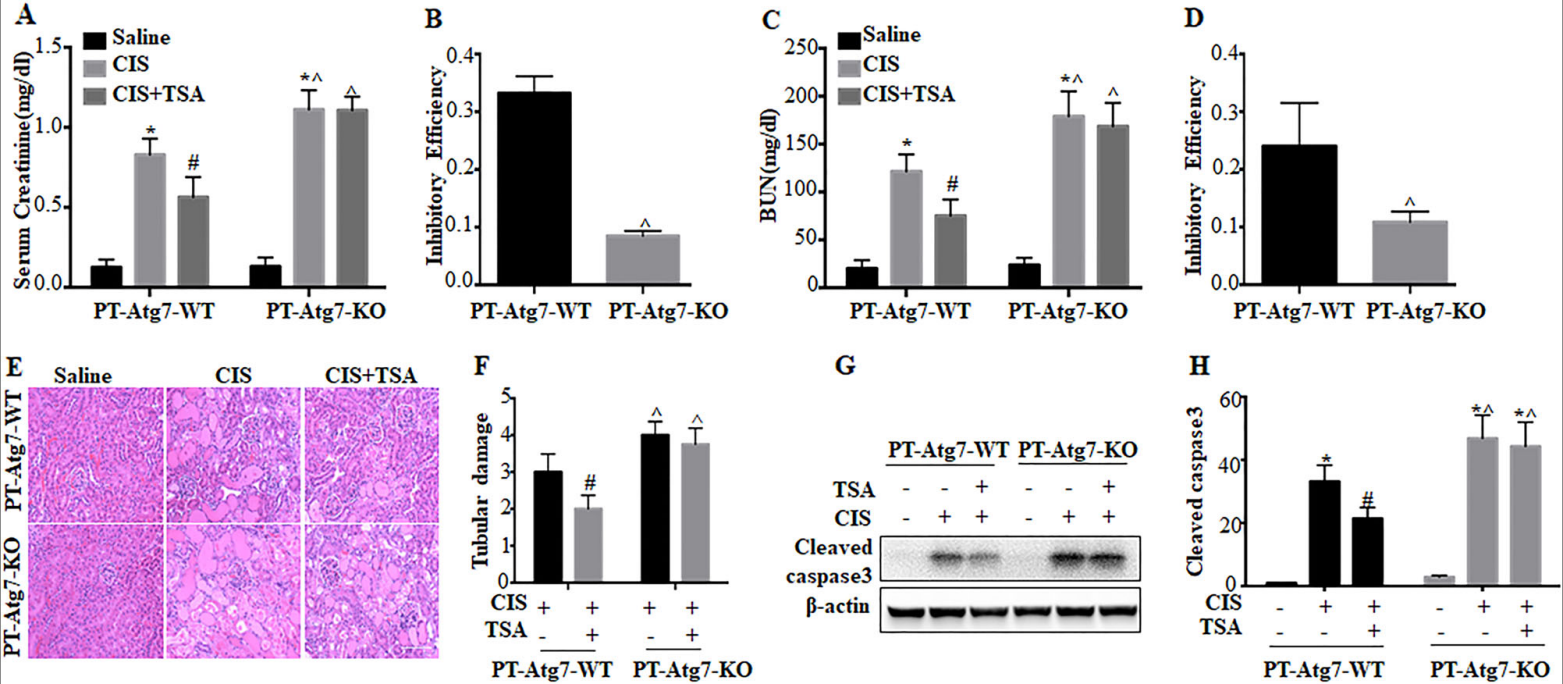
The renoprotective effect of TSA in cisplatin-induced AKI is lost in PT-*Atg7* KO mice. PT-*Atg7* WT and PT-*Atg7* KO mice were injected with saline or a single dose of cisplatin (30 mg/kg, i.p.) in the absence (−) or presence (+) of TSA (1 mg/kg, i.p., daily injection). Three days after treatment, blood samples and kidneys were collected for biochemical and histological analyses. **a** Serum creatinine. **b** Efficiency of TSA reducing serum creatinine (cisplatin only vs cisplatin+TSA). **c** BUN. **d** Efficiency of TSA reducing BUN (cisplatin only vs cisplatin+TSA). Data in **a**–**d** are expressed as mean ± SD. *P < 0.05, significantly different from the saline group. ^#^*P* < 0.05, significantly different from the cisplatin-only group. ^*P < *0.05, significantly different from the corresponding group of PT-*Atg7* WT mice. **e** Representative images of kidney H–E staining (×200). Scale bar: 100 μm. **f** Pathological score of tubular damage. Data are expressed as mean ± SD. ^#^*P* < 0.05, significantly different from the cisplatinonly group. ^*P* < 0.05, significantly different from the PT-Atg7-WT group. **g** Representative immunoblots. **h** Densitometric analysis of cleaved caspase 3 signals. Data are expressed as mean ± SD. *P < 0.05, significantly different from the saline group. ^#^*P < *0.05, significantly different from the cisplatin-only group. ^*P < *0.05, significantly different from the PT-Atg7-WT group

We further monitored kidney injury. In WT mice, cisplatin induced increases in both serum creatinine and blood urea nitrogen (BUN) (Fig. 9a, c: PT-*Atg7* WT, CIS). TSA partially but significantly suppressed the loss of renal function. Serum creatinine and BUN were reduced from 1.9 and 158 mg/dl in the cisplatin group to 1.2 and 119 mg/dl in the cisplatin+TSA group, respectively (Fig. 9a, c: PT-*Atg7* WT, CIS vs CIS+TSA). Consistently, TSA also ameliorated cisplatin-induced tubular damage in renal cortex and out medulla, as indicated by histological examination. Cisplatin-treated mice had a tubular damage score of 3, which was reduced to 1.9 by TSA (Fig. 9e, f: PT-*Atg7* WT, CIS vs CIS+TSA). Cisplatin-induced caspase activation was also attenuated by TSA (Fig. 9g, h: PT-*Atg7* WT, CIS vs CIS+TSA). We then determined the effects of TSA in PT-*Atg7* KO mice with impaired autophagy in proximal tubules. Consistent with previous work^34^, PT-*Atg7* KO mice developed more severe kidney injury following cisplatin treatment than their WT littermates (Fig. 9a, c, e–h: WT vs KO, CIS). Notably, TSA only had marginal inhibitory effects on cisplatin-induced renal function loss in PT-*Atg7* KO mice (Fig. 9a, c: PT-*Atg7* KO, CIS vs CIS+TSA). The inhibition efficiency of TSA on serum creatinine and BUN in WT mice was 25–35%, which was decreased to ~10% in PT-*Atg7* KO mice (Fig. 9b, d). Moreover, TSA did not have obvious effects on cisplatin-induced tubular cell injury and death in PT-*Atg7* KO mice (Fig. 9e–h: PT-*Atg7* KO, CIS vs CIS+TSA). These results, together with the chloroquine test (Fig. 7), provide compelling in vivo evidence that TSA protects against cisplatin-induced AKI by activating autophagy in proximal tubules.

### TSA activates AMPK and inactivates mammalian target of rapamycin (mTOR) during cisplatin treatment of RPTC cells and C57Bl/6 mice

**Fig. 10:**
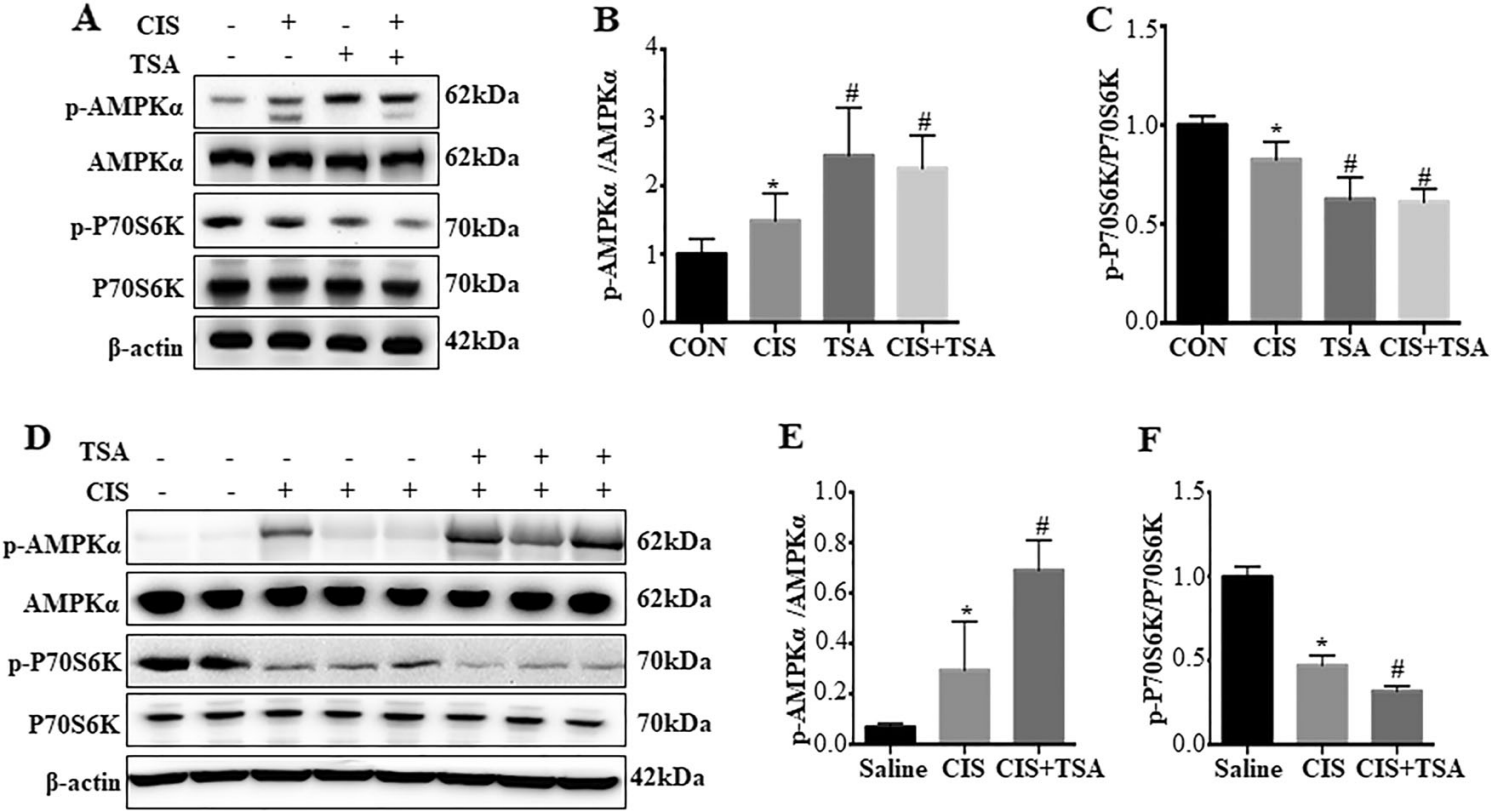
TSA activates AMPK and inactivates mTOR during cisplatin treatment of RPTC cells and C57Bl/6 mice. **a**–**c** RPTC cells were untreated or treated with cisplatin (20 μM) only, TSA (0.1 μM) only, or cisplatin+TSA for 20 h. After treatment, cells were collected for immunoblot analysis of p-AMPK, AMPK, p-P70S6K, and P70S6K. β-Actin was used as a loading control. **a** Representative immunoblots. **b**, **c** Densitometric analysis of p-AMPK and p-P70S6K signals after normalization with AMPK and P70S6K, respectively. Data are expressed as mean ± SD. *P < 0.05, significantly different from the control group. ^#^*P* < 0.05, significantly different from the cisplatin-only group. **d**–**f** C57BL/6 mice were injected with saline or a single dose of cisplatin (30 mg/kg, i.p.) in the absence (−) or presence (+) of TSA (1 mg/kg, i.p., daily injection). Kidneys were collected 3 days after treatment for immunoblot analysis of p-AMPK, AMPK, p-P70S6K, and P70S6K. β-Actin was used as a loading control. **d** Representative immunoblots. **e**, **f** Densitometric analysis of p-AMPK and p-P70S6K signals after normalization with total AMPK and P70S6K, respectively. Data are expressed as mean ± SD. *P < 0.05, significantly different from the saline group. ^#^*P* < 0.05, significantly different from the cisplatin-only group

Autophagy involves a complex molecular machinery that is regulated at various levels by multiple signaling pathways^24,25^. To gain initial insights into the mechanism by which TSA activates autophagy in proximal tubular cells, we examined AMPK and mTOR, two well-recognized autophagy regulatory pathways^24,25,37^. Cisplatin induced phosphorylation/activation of AMPK in RPTC cells, which was accompanied with marginal inactivation of mTOR as indicated by decrease in phosphorylated P70S6K (p-P70S6K) (Fig. 10a–c). TSA, either used alone or with cisplatin, significantly increased p-AMPK and decreased p-P70S6K, suggesting AMPK activation and mTOR inactivation by TSA (Fig. 10a–c). Similar effects of TSA were observed in mouse kidney tissues (Fig. 10d–e). These results suggest that TSA may enhance autophagy in renal tubular cells to protect kidneys by activating AMPK and suppressing mTOR.

## Discussion

Recent studies have demonstrated the therapeutic effects of HDACis in several experimental models of kidney diseases, but the underlying mechanisms remain largely unclear. The current study has further confirmed the protective effects of HDACi in cisplatin-induced nephrotoxicity. Importantly, we show that TSA and SAHA, two classical HDACis, can stimulate autophagy in RPTCs and kidneys during cisplatin treatment. Inhibition of autophagy either by chloroquine or by *Atg7* knockout in proximal tubular cells led to the attenuation of the renoprotective effects of TSA, supporting a critical role of autophagy in the protective action of TSA. Mechanistically, TSA stimulated AMPK and inactivated mTOR during cisplatin treatment, suggesting that HDACis may enhance autophagy by activating AMPK and inhibiting mTOR, two major regulators of autophagy^24,25,37^.

Arany et al.^7^ demonstrated the cytoprotective effects of TSA against cisplatin-induced apoptosis in mouse RPTCs. We further verified protective effects of SAHA and TSA in rat RPTCs^8^. Notably, these agents also increased cell recovery from cisplatin cytotoxicity, indicating a long-term pro-survival effect^8^. Mechanistically, we showed that SAHA and TSA could block the DNA damage response during cisplatin treatment, resulting in the suppression of p53 activation, a major signaling pathway for cisplatin-induced tubular cell apoptosis^8^. Ramesh and colleagues^9^ further revealed the anti-inflammation effects of HDACis in cisplatin nephrotoxicityby transcriptional induction of activated microglia/macrophage WAP domain protein. Our current study has identified autophagy as a new mechanism responsible for HDACi-mediated renoprotection during cisplatin nephrotoxicity. In our experiments, both TSA and SAHA induced autophagy in proximal tubular cells (Fig. 1, Supplementary Figure 1). TSA further enhanced autophagy during cisplatin-treatment of proximal tubular cells and kidneys (Fig. 2 and Fig. 6). These morphological and biochemical analyses confirmed autophagy induction and enhancement by HDACis at steady-state levels and, more importantly, revealed the dynamic regulation of autophagy by these agents. To define the role of autophagy, we used both pharmacological and genetic approaches. In cultured proximal tubular cells, impairment of autophagy either by chloroquine or *Atg7* knockout significantly attenuated the inhibitory effects of TSA on cisplatin-induced apoptosis (Figs. 3 and 5). Consistently, TSA lost its renoprotective effects in mice when autophagy was blocked by chloroquine or *Atg7* ablation from kidney proximal tubules (Figs. 7 and 9). These results unequivocally demonstrate the importance of autophagy to the protective effects of HDACi in cisplatin nephrotoxicity. Interestingly, impairment of autophagy may also delays kidney recovery from ischemia–reperfusion injury^38^, suggesting a role of autophagy in cell or tissue recovery from injury.

A few recent studies have eluded the possible regulation of autophagy by HDACi. Patschan et al. demonstrated that SAHA could abrogate the autophagy inhibitory effect of transforming growth factor-β in endothelial progenitor cells^39^. Khan et al. showed that HDACi may prevent the decline of podocyte autophagy in diabetic models and attenuate podocyte injury as well as proteinuria^40^. SAHA was also shown to increase autophagic flux in rabbit myocardium and protect against ischemia/reperfusion injury^41^, while TSA may promote autophagy in neurons and ameliorate neuronal apoptosis and brain injury induced by subarachnoid hemorrhage in rats^42^. Despite these studies, the role of autophagy in the effects of HDACis remains unclear. Using pharmacologic and genetic inhibitory approaches, our current study has shown convincing evidence that autophagy is critical to the renoprotective effects of HDACi in cisplatin nephrotoxicity.

As a promising class of anticancer agents, HDACi have synergistic or additive tumor-killing effects with many other anticancer drugs such as cisplatin^43^. Using HDACi to prevent renal damage without affecting the efficacy of cisplatin in cancer treatment appears to be a very attractive therapeutic strategy. In this regard, our previous study showed that SAHA, while reducing cisplatin-induced apoptosis in kidney tubular cells, could enhance apoptosis during cisplatin treatment of HCT116 colon cancer cells^8^, supporting the possibility of in vivo use of HDACis for renoprotection while enhancing the tumor-killing effects of cisplatin. Although the death-promoting mechanisms of SAHA in HCT116 cancer cells remain unclear, the results indicate that normal cells and cancer cells may respond differently to HDACis^8^. Indeed, in cancer models of hepatocellular carcinoma, endometrial stromal sarcoma, pancreatic cancer, and ovarian cancer, HDACis can induce autophagy to elicit both apoptotic and non-apoptotic cell death^3^. Given these findings, HDACi-induced autophagy in normal renal tubular cells may represent a new kidney-specific protective strategy during cisplatin-mediated chemotherapy. Kidney tubular cells may have significantly higher endonuclease and caspase activities than cancer cells^44,45^, which may contribute to the sensitivity of kidney tissues to HDACi protection.

The mechanisms whereby HDACis regulate autophagy remain largely unclear. HDAC1, HDAC2, HDAC6, Sirt3, and Sirt6 were recently shown to increase at 24 h of cisplatin nephrotoxicity, while HDAC1, HDAC2, HDAC3, HDAC6, Sirt3, and Sirt4 were induced at 72 h^9^. However, it remains unclear which HDAC(s) mediate cisplatin nephrotoxicity. Provided the role of HDAC in regulating acetylation, several ATG proteins may be subjected to the regulation by acetylation/deacetylation^46,47^. To understand how HDACis activate autophagy in proximal tubular cells, we examined the effects of TSA on AMPK and mTOR, two well-recognized autophagy regulator. We found that TSA could enhance AMPK activation, while blocking mTOR activation, in cisplatin nephrotoxicity (Fig. 10). In view of the opposite functions of AMPK and mTOR in autophagy regulation, it is suggested that HDACis may activate autophagy during cisplatin nephrotoxicity at least partially through AMPK and mTOR.

## Materials and methods

### Reagents and antibodies

TSA was purchased from Enzo Life Sciences. Carbobenzoxy-Asp-Glu-Val-Asp-7-amino-4-trifluoromethyl coumarin (DEVD-AFC) and 7-amino-4-trifluoromethyl coumarin (AFC) were from Enzyme Systems Products (Livermore, CA). Unless indicated, all other reagents including cisplatin and chloroquine were purchased from Sigma (St. Louis, MO). The following primary antibodies were used: anti-LC3B from Novus Biologicals (Littleton, CO); anti-ATG7, anti-β-actin and anti-cyclophilin B from Abcam; and anti-cleaved caspase3, anti-AMPK, anti-phospho-AMPK (Thr172), anti-P70S6K, anti-phospho-P70S6K (T389) from Cell Signaling Technology (Danvers, MA). All secondary antibodies for immunoblot analysis were from Thermo Scientific (Rockford, IL).

### Animals

C57BL/6 mice were purchased from Jackson Laboratory (Bar Harbor, ME). Using Cre-loxP technology, a conditional KO mouse model with *Atg7* specifically deleted from renal proximal tubules (PT-*Atg7* KO) was recently generated and characterized by our laboratory^34,48^. The autophagy reporter mice expressing RFP-GFP-LC3 transgene under the control of a CAG promoter (CAG-RFP-GFP-LC3 mice) were originally provided by Dr. Joseph A. Hill at University of Texas Southwestern Medical Center (Dallas, TX)^36^. All animals were maintained in a pathogen-free facility at Charlie Norwood VA Medical Center under 12/12-h light/dark pattern with free access to water and food. All animal experiments were carried out according to a protocol approved by the Institutional Animal Care and Usage Committee in Charlie Norwood VA Medical Center.

### Mouse model of cisplatin-induced AKI and TSA treatment

Male mice aged 8–12 weeks were used in this study. For cisplatin injury, mice were intraperitoneally (i.p.) injected with a single dose of cisplatin at 30 mg/kg, while control animals were injected with a comparable volume of saline^49^. To test the effect of chloroquine, 60 mg/kg chloroquine was injected (i.p.) 1 h prior to cisplatin administration and then daily after cisplatin treatment. To test the effect of TSA, 1 mg/kg TSA was injected (i.p.) 2 days prior to and daily after cisplatin administration.

### Analysis of autophagy dynamics by a tandem mRFP-GFP-LC3 reporter in transfected cells and in transgenic mice

The dynamic process of autophagy (autophagosome formation and maturation to autolysosomes) was analyzed in cultured proximal tubular cells expressing mRFP-GFP-LC3 or in CAG-RFP-GFP-LC3 mice as described in our recent work^35^. The rationale of this method is that acid-sensitive GFP is quenched in the low pH lysosomal environment, whereas acid-insensitive RFP is more stable and maintained. Thus co-localization of RFP fluorescence with GFP in a particle indicates an autophagosome, and a RFP-only signal is considered an autolysosome^36,50^. For in vitro experiments, cultured proximal tubular cells were transiently transfected with mRFP-GFP-LC3 (ptfLC3, Addgene plasmid 21074). After treatment, the cells were fixed with 4% paraformaldehyde for fluorescence microscopy (Zeiss 780 upright confocal microscope). For quantitative analysis, approximately 100 transfected cells from 10 to 20 random fields (×630) were analyzed in each condition. The numbers of GFP-LC3 puncta per cell and RFP-LC3 puncta per cell were counted separately using ImageJ. The number of autophagosomes was indicated by GFP dots and the number of autolysosomes was obtained by subtracting GFP dots from RFP dots. The number of autolysosomes was further divided by the total number of RFP dots to indicate the autophagic flux rate. For in vivo experiments, after treatment CAG-RFP-GFP-LC3 mice were perfused fixed with 4% paraformaldehyde. Kidneys were further fixed overnight with the same fixative, balanced with 30% sucrose, and embedded in Optimal Cutting Temperature compound for cryo-section and confocal microscopy. For each section, 8–10 fields (×630) were selected randomly and quantitative analysis was performed by the method described above in cultured cells.

### Renal function

Renal function was determined by BUN and serum creatinine measurements using commercial kits from Stanbio Laboratory (Boerne, TX). In brief, blood samples were collected for coagulation and centrifugation at room temperature to collect serum. For BUN, the reaction was conducted at 100 °C for 12 min and the absorbance at 520 nm was recorded by the end of reaction. For serum creatinine, samples were added to a pre-warmed (37 °C) reaction mixture and the absorbance at 510 nm was monitored kinetically at 20 and 80 s of reaction. BUN and creatinine levels (mg/dl) were then calculated based on standard curves.

### Histological examination

Kidney tissues were harvested, fixed with 4% paraformaldehyde, embedded in paraffin, and sectioned at 4 µm. H–E staining was performed using standard procedures and renal tubules with the following histopathological changes were considered injured: loss of brush border, tubular dilation and disruption, cast formation, and cell lysis. Tissue damage was examined in a blind manner and scored by the percentage of damaged tubules: 0, no damage; 1, <25%; 2, 25–50%; 3, 50–75%; 4, >75%.

### Immunohistochemical staining of LC3B

Kidneys were fixed in 4% paraformaldehyde overnight and processed for routine embedding in paraffin. After rehydration, antigen retrieval was performed by incubation with 1 mM EDTA (pH 8.0) at 95–100 °C for 1 h. After subsequently incubated with 0.03% H_2_O_2_ to block endogenous peroxidase activity and with a buffer containing 2% bovine serum albumin, 0.2% milk, 2% normal donkey serum, and 0.8% Triton X-100 to reduce non-specific binding, the slides were exposed to anti-LC3B antibodies at 4 °C overnight. Negative controls were done by replacing the primary antibody with antibody diluent. After incubation with avidin–biotin blocking reagent (Vector Laboratories, SP-2001), the slides were exposed to 1:500 biotinylated donkey anti-rabbit secondary antibody (Millipore, AP) for 1 h at room temperature. Following signal amplification with Tyramide Signal Amplification Biotin System (Perkin Elmer, NEL700A001KT), the sections were incubated with a VECTASTAIN® ABC kit (Vector Laboratories, PK-6100) and signals were developed with a DAB kit (Vector Laboratories). For quantification of LC3B staining, 10–20 fields (×400) were randomly selected from each slide and the number of LC3B puncta per proximal tubule was evaluated using ImageJ.

### Cell lines

Immortalized RPTC line was originally obtained from Dr. Ulrich Hopfer (Case Western Reserve University, Cleveland, OH) and maintained in Dulbecco's Modified Eagle's Medium/F-12 medium supplemented with 10% fetal bovine serum and growth factors^51^. Stable floxed control (*Atg7* WT) and *Atg7* KO MPTC lines (MPTC) were generated in our recent study^35^. Briefly, primary kidney proximal tubular cells were isolated from 5-week-old *Atg7*-loxP mice. The cells were cultured for immortalization by transfecting with SV40 larger T-antigen. The immortalized proximal tubular cells were then infected with retroviruses expressing Cre-PURO-IRES-GFP (pMSCV-Cre-PIG, Addgene plasmid 50395) or control viruses to establish stable *Atg7* WT and *Atg7* KO cell lines.

### Cisplatin and TSA treatment of RPTCs

Cells were plated in 35-mm dishes to reach ~ 90% confluence by next day. To induce apoptosis, cells were incubated with 20 μM cisplatin for 20 h. TSA was given either alone at different concentrations (0.05, 0.1, 0.2 μM) or together with cisplatin at a concentration of 0.1 μM. In some experiments, cells were also treated with cisplatin and TSA in the presence of 20 μM chloroquine. After treatment, cells were monitored morphologically or harvested for biochemical analyses. For cell lysis, both floating and adherent cells were collected.

### Examination of apoptosis

Apoptosis was examined by morphology and caspase activity by standard methods. Morphologically, cells were stained with 10 μg/ml Hoechst 33342 to examine cellular and nuclear morphology by phase contrast and fluorescence microscopy, respectively. For each condition, four fields with ~200 cells per field were randomly selected and cells with typically apoptotic characterizations were counted to estimate apoptosis percentage. To measure caspase activity, cells were extracted with 1% Triton X-100 and 20 μg protein were added to enzymatic reactions containing 50 μM DEVD-AFC, a fluorogenic peptide substrate of caspases. After 1 h incubation at 37 °C, fluorescence was measured at excitation 360 nm/emission 530 nm. Based on a standard curve constructed with free AFC, the fluorescence reading was converted into the nanomolar amount of liberated AFC per mg protein to indicate caspase activity.

### Immunoblot analysis

Whole cell or tissue lysates from renal cortex and out medulla were extracted in 2% sodium dodecyl sulfate (SDS) buffer (62.5 mM Tris-HCl, pH 6.8, 2% SDS, 10% glycerol) containing protease inhibitor cocktail (Sigma-Aldrich) and Benzonase nuclease (EMD Millipore). Protein concentration was estimated with the BCA Protein Assay Kit (Thermo Scientific). Equal amounts of protein were loaded in each lane and separated by SDS-polyacrylamide gel electrophoresis. After transfer to polyvinylidene difluoride membranes, blots were blocked in 5% milk, probed with primary and secondary antibodies, and visualized with an enhanced chemiluminescence kit. Either β-actin or cyclophilin B was used to monitor protein loading and transferring.

### Statistics

Qualitative data including immunoblots and cell images are representatives of at least three experiments. Quantitative data were expressed as means ± SD. Statistical analysis was conducted using the GraphPad Prism software. Statistical differences in multiple groups were determined by multiple comparisons with analysis of variance followed by Tukey’s post-tests. Statistical differences between two groups were determined by two-tailed unpaired or paired Student's *t*-test. *P* < 0.05 was considered significantly different.

## Electronic supplementary material

Supplementary Figure 1

Supplementary Figure 1 Legend
