# Supplementary figures and images for: Histone deacetylase inhibitors protect against cisplatin-induced acute kidney injury by activating autophagy in proximal tubular cells
Source: Cell Death Dis. 2018 Feb 23;9(3):322. doi: 10.1038/s41419-018-0374-7 (PMC5833747; doi:10.1038/s41419-018-0374-7)

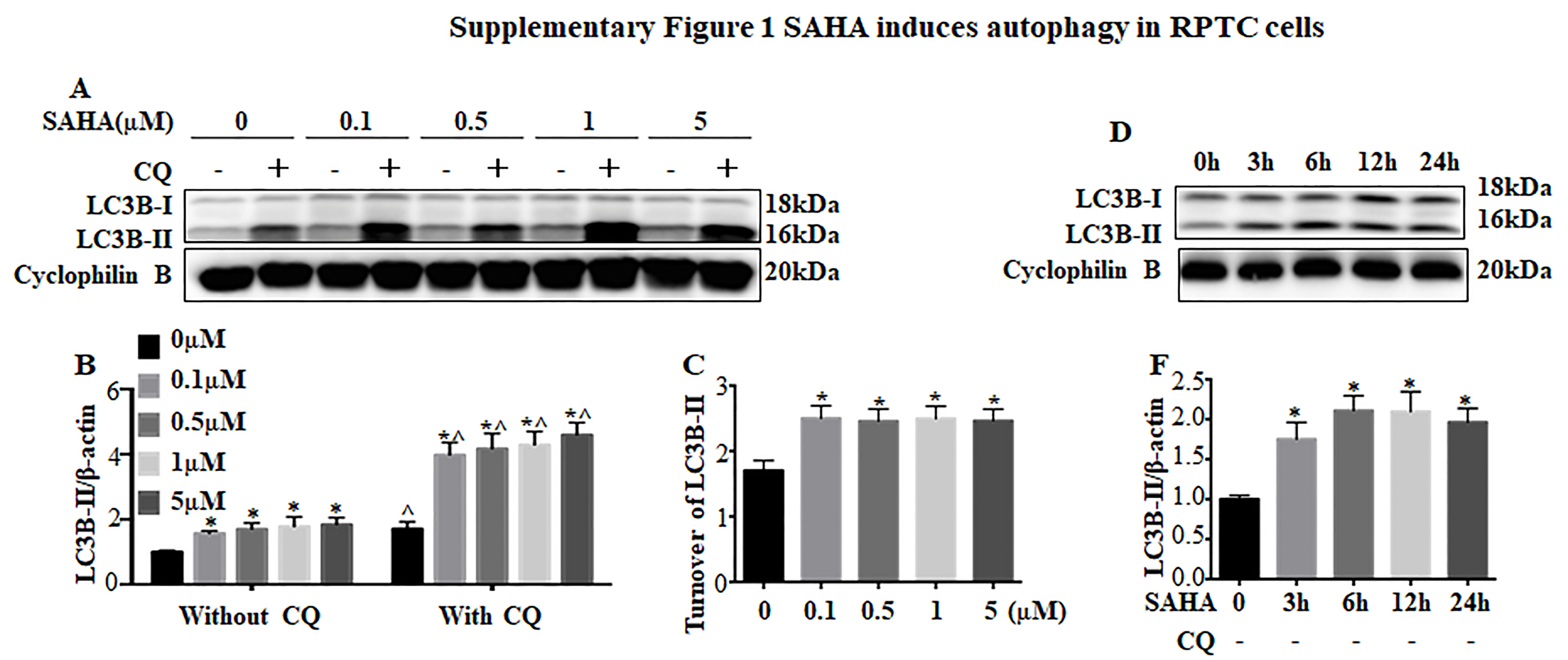

Supplement: Supplementary file 1 — Supplementary Figure 1 [file 41419_2018_374_MOESM1_ESM.tif]
