# Supplementary material for: Histone deacetylase inhibitors protect against cisplatin-induced acute kidney injury by activating autophagy in proximal tubular cells
Source: Cell Death Dis. 2018 Feb 23;9(3):322. doi: 10.1038/s41419-018-0374-7 (PMC5833747; doi:10.1038/s41419-018-0374-7)
Supplement: Supplementary file 2 — Supplementary Figure 1 Legend [file 41419_2018_374_MOESM2_ESM.docx]

**Supplementary Figure 1 SAHA induces autophagy in RPTC cells. (A-C)** RPTC cells were subjected to the treatment with indicated concentrations of SAHA for 24 h in the absence or presence of 20 μM chloroquine. **(D** and **E)** RPTC cells were treated with 1 μM SAHA for the indicated times. After treatment, whole cell lysates were collected for immunoblot analysis of LC3B. Cyclophilin B was used as a loading control. **(A** and **D)** Representative images of LC3B immunoblot. **(B, C, E)** Densitometric analysis of LC3B signals. After normalized with cyclophilin B, the protein signal of control was arbitrarily set as 1, and the signals of other conditions were normalized with control to calculate fold changes. Data are expressed as mean±SD . * *P*<0.05, significantly different from control group.
